# Generating a Non-Integrating Human Induced Pluripotent Stem Cell Bank from Urine-Derived Cells

**DOI:** 10.1371/journal.pone.0070573

**Published:** 2013-08-05

**Authors:** Yanting Xue, Xiujuan Cai, Linli Wang, Baojian Liao, Hui Zhang, Yongli Shan, Qianyu Chen, Tiancheng Zhou, Xirui Li, Jundi Hou, Shubin Chen, Rongping Luo, Dajiang Qin, Duanqing Pei, Guangjin Pan

**Affiliations:** 1 CAS Key Laboratory of Regenerative Biology, South China Institute for Stem Cell Biology and Regenerative Medicine, Guangzhou Institutes of Biomedicine and Health, Chinese Academy of Sciences, Guangzhou, Guangdong, China; 2 School of Life Sciences, University of Science and Technology of China, Hefei, Anhui, China; 3 Guangdong Provincial Key Laboratory of Stem cell and Regenerative Medicine, South China Institute for Stem Cell Biology and Regenerative Medicine, Guangzhou Institutes of Biomedicine and Health, Chinese Academy of Sciences, Guangzhou, Guangdong, China; Leiden University Medical Center, Netherlands

## Abstract

Induced pluripotent stem cell (iPS cell) holds great potential for applications in regenerative medicine, drug discovery, and disease modeling. We describe here a practical method to generate human iPS cells from urine-derived cells (UCs) under feeder-free, virus-free, serum-free condition and without oncogene *c-MYC*. We showed that this approach could be applied in a large population with different genetic backgrounds. UCs are easily accessible and exhibit high reprogramming efficiency, offering advantages over other cell types used for the purpose of iPS generation. Using the approach described in this study, we have generated 93 iPS cell lines from 20 donors with diverse genetic backgrounds. The non-viral iPS cell bank with these cell lines provides a valuable resource for iPS cells research, facilitating future applications of human iPS cells.

## Introduction

Human somatic cells can be reprogrammed back into pluripotent state as induced pluripotent stem cells that can undergo unlimited self-renewal while maintaining the potential to differentiate into all three germ layers. Human iPS cells hold great potential to be used for personalized regenerative medicine, drug discovery and disease modeling, for circumventing ethical issues [1]. To date, human iPS cells had been successfully generated from different tissue sources, such as skin fibroblasts [2], [3], extra-embryonic tissues [4] and blood cells [5], although the reprogramming efficiencies varied much for different cell types. Moreover, the virus-based approach used in most of these reports raised the safety concerns, which hampered their further applications. To enable the application of iPS technology, it is essential to develop and optimize a practical approach to generate iPS cells under a feeder-free, virus-free and serum-free condition. To date, several non-viral approaches had been reported to generate mouse and human iPS cells, by using adeno-virus [6], plasmids [7], purified proteins [8], [9], synthesized mRNA [10], [11], [12]. However, these approaches remain inefficient or laborious. We and others had previously described that epithelia-like cells could be isolated from human urine and could be further reprogrammed into iPS cells with high efficiency by using virus-based approach [13], [14], [15], [16]. Human UCs can be conveniently obtained through non-invasive method, thus representing an ideal cell source for iPS cell generation. Here, in this report, we had examined whether isolation of UCs to generate iPS cells can be applied in a large population with different genetic background. We firstly describe an efficient method to reprogram human UCs without using virus, serum, feeder and without oncogene *c-MYC*. By using this method, we have been generating a non-viral human iPS cell bank from donors in different ages and various genetic and disease backgrounds. Our non-integrating human iPS cell bank, enables comprehensive study of iPS cells from different origins, and also provides a valuable source for disease modeling and mechanical analysis.

## Results and Discussion

**Figure 1:**
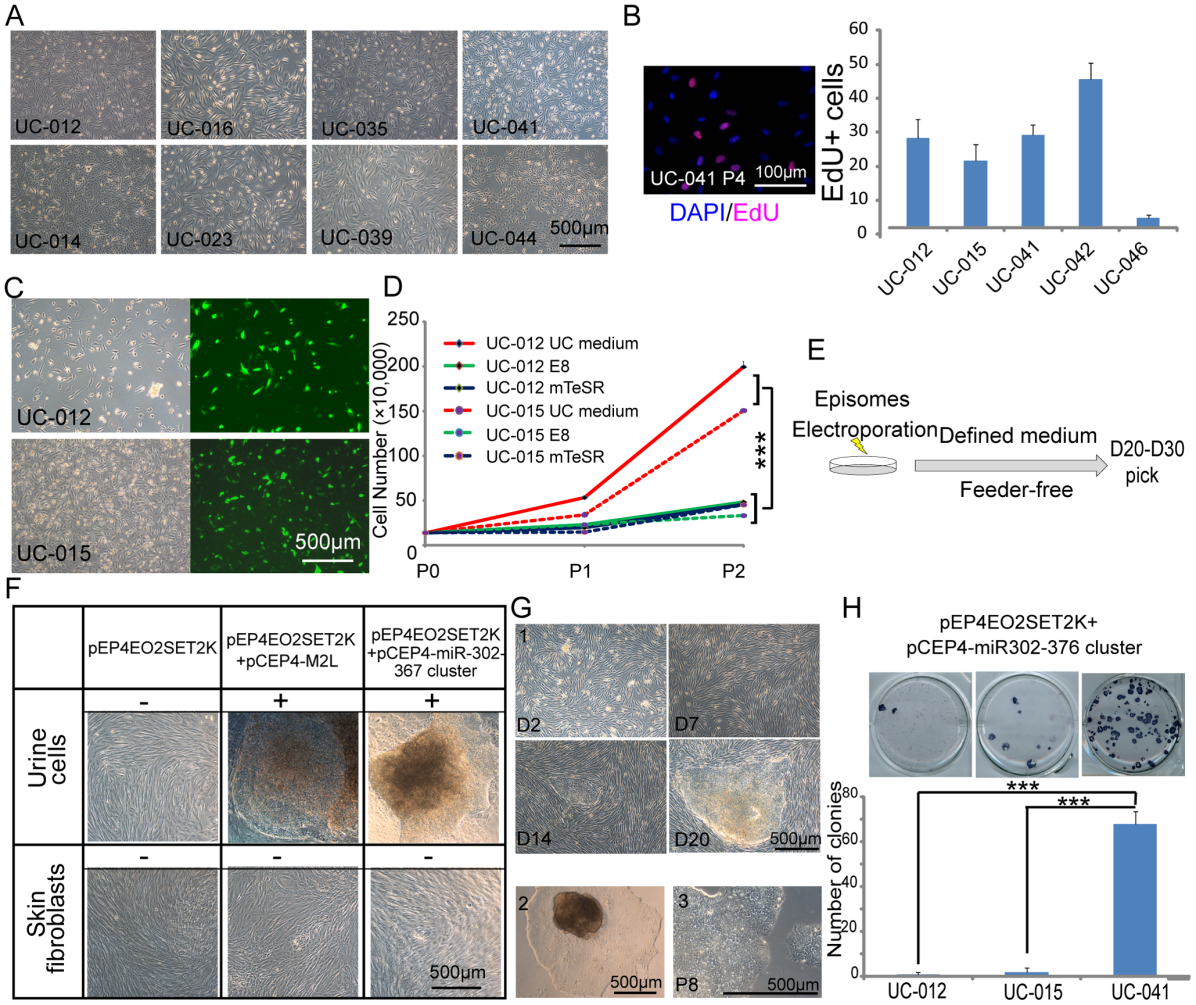
Optimization of a method to generate non-integrated iPS cells from UCs. A. UCs from healthy (UC-012) and diseased donors (listed in Table 1 and Table 2). B. Left: EdU imaging of representative UC. Right: EdU positive percentages of 5 UCs. Error bars are standard deviation of the mean, n = 3. C. Phase contrast and fluorescent photographs of UC-012 and UC-015 electroporation with episomal plasmid pCEP4-EGFP and cultured for 24 h in UC medium. D. Growth curves of UC-012 and UC-015 in UC medium, defined medium E8 and mTeSR1, respectively. *** indicates *P*<0.001. E. Schematic representation of iPS cell generation. The defined medium can be either mTeSR1 or E8. F. Different reprogramming factor combinations for iPS cell generation of UCs and skin fibroblasts from the same donor. −: failure, +: success. G. Representative phase contrast photographs of a successful iPS cell generation process: (1) Emerging iPS cell colony generating by our optimized method in E8 from UC-012 at different time points. (2)A picked colony. (3) The established iPS cell line from this colony. P8: passage 8. H. Top: AP staining of iPS cells generated from UC-012, UC-015 and UC-041 in 6 well plates. I. Bottom: reprogramming efficiencies of UC-012, UC-015 and UC-041. n = 3.

We have easily recruited people for urine donation because urine collection is non-invasive and routinely carried out for clinical diagnosis (55 out of 60 surveyed people accepted to donate urine sample. Fig. S1A). With consent form signed by the donors, we have collected and established 46 UC lines (Table 1 and Table 2) from 50–200 ml middle stream of the micturition. These donors include people with different ages (from 2 to 73 years old) and various disease genetic backgrounds. For all the donated urine samples examined so far, we could successfully isolate and expand epithelia cells from more than 90% of them, demonstrating that our approach for isolating UCs can be universally applied for most people (Fig. 1A). For these 46 established UC lines, 19 of them were collected from healthy people and 27 from patients that were diagnosed clinically with different diseases, such as hereditary blood disorders (hemophilia and β-thalassemia), autoimmune diseases, nervous neurological disorders (amyotrophic lateral sclerosis ALS, and myotonic dystrophy *et al.*). For the genetic diseases, we confirmed the mutations of β-thalassemia in 2 donors (Fig. S1B). Potential mutations of Alport’s syndrome and ALS were not detected in the samples (5 genes known to cause ALS and 7 mutation sites of *COL5A4* causing Alport’s syndrome were listed in Table S1 and Table S2) because they are complicated genetic diseases. We also analyzed the proliferation percentage of UCs by EdU assay (Fig. 1B). Most of these UC lines proliferated well and could be expanded for more than 5 passages. In some cases (2 out of 46), we had to recollect the urine samples in order to get enough cells for further culture.

**Table 1:** List of UiPSCs generated from UCs.

Cell code	Geneticbackgrounds	Age (y)	M/F*	Efficiency	Coloniespicked	Karyotype**	qPCR	Non-integrating	IF/FACS	Teratoma
UC-001	Alports disease	45	M	0.0026%	8	4/8	3/8	3/8	5/8	2/8
UC-002	Hemophilia B	2	M	0.0013%	15	3/15	2/15	3/15	2/15	ND
UC-003	Healty	25	M	ND	3	2/3	3/3	3/3	2/3	ND
UC-004	Hemophilia A	10	M	0.0006%	4	1/4	1/4	1/4	1/4	ND
UC-005	Hemophilia A	2.5	M	0.0024%	7	2/7	2/7	2/7	2/7	2/7
UC-006	Hemophilia A	21	M	ND	6	1/6	1/6	1/6	1/6	ND
UC-007	Hemophilia A	22	M	ND	2	1/2	1/2	1/2	1/2	ND
UC-008	Healty	27	M	0.0001%	1	1/1	1/1	1/1	1/1	1/1
UC-009	β-thalassemia	3	M	ND	8	2/8	2/8	2/8	2/8	ND
UC-010	Healty	60	M	0.0002%	2	1/2	1/2	1/2	1/2	ND
UC-011	Hemophilia A	12	M	ND	5	5/5	1/5	1/5	1/5	2/5
UC-012	Healty	25	M	0.0003%	5	2/5	2/5	2/5	1/5	1/5
UC-013	Healty	26	M	0.001%	3	1/3	1/3	1/3	1/3	ND
UC-014	CMT	42	M	0.013%	4	2/4	2/4	2/4	1/4	ND
UC-015	Healty	25	M	0.0007%	4	3/4	3/4	4/4	2/4	1/4
UC-027	Healty	26	F	ND	2	1/8	2/8	2/8	2/8	ND
UC-040	β-thalassemia	12	M	ND	2	2/2	ND	ND	ND	ND
UC-041	β-thalassemia(carrier)	28	F	0.015%	6	2/6	2/6	2/6	1/6	1/6
UC-044	ALS	54	M	ND	6	ND	ND	ND	ND	ND
UC-046	Healty	70	M	0.007%	4	1/4	ND	ND	ND	ND

**Table 2:** List of other UC lines.

Cell code	Genetic backgrounds	M/F*	Age(year)
UC-016	Hemophilia A	M	12
UC-017	Hemophilia A	M	7
UC-018	Hemophilia A	M	2.5
UC-019	Hemophilia A	M	6
UC-020	Hemophilia A	M	2.5
UC-021	Hemophilia A	M	50
UC-022	Hemophilia A	M	10
UC-023	Hemophilia A	M	23
UC-024	Healthy	F	22
UC-025	Healthy	F	25
UC-026	Healthy	M	25
UC-028	Healthy	M	25
UC-029	Healthy	M	25
UC-030	Healthy	F	31
UC-031	Healthy	M	31
UC-032	Healthy	M	29
UC-033	Amyotrophic lateral sclerosis	M	73
UC-034	Healthy	M	69
UC-035	Systemic lupus erythematosus	F	
UC-036	β-thalassemia	M	5
UC-037	Alport’s syndrome	F	39
UC-038	Amyotrophic lateral sclerosis	M	57
UC-039	Parkinson Disease	M	
UC-042	Healthy	M	23
UC-043	Healthy	M	29
UC-045	β-thalassemia(carrier)	F	54

* M: male F:female.

** 4/8:4 out of 8.

To obtain non-integrating iPS cells from these UC lines, we chose episomal system to deliver the reprogramming factors. Episome, which derived from EBV EBNA/OriP system to enable the replication of transfected plasmid in eukaryotic cells, had been used in iPS cell generation from neonatal fibroblasts and CD34+ hematopoietic progenitor cells in different laboratories [17], [18], [19]. Except specific donor cell type human adult adipose tissue-derived stem cells (AdSC) [20], most of these reports included oncogene *c-MYC* as reprogramming factor, raising risks in maintaining genomic stability during iPS generation [19], [20] In addition, some of them used serum and mouse feeder cells for reprogramming [17], [18]. Therefore, we sought to reprogram human UCs through episomal system without using serum, feeders and *c-MYC*. To test the episomal system in UCs, we transfected an episomal vector encoding EGFP into these cells through electroporation. We showed that EGFP expression efficiency was remarkable, about 35% cells were positive for EGFP expression (Fig. 1C). Next, we found that UCs could survive and grow in serum-free media mTeSR1 and E8 which were used to maintains human ES or iPS cells [21], [22], albeit slower than in their optimal medium (Fig. 1D). These data suggested that it was possible to use episomal system and defined serum-free medium for iPS generation with UCs as illustrated in Figure 1E. Because involvement of oncogene *c-MYC* during reprogramming might increase the risk of genomic toxicity [23], we tried to omit it by using *OCT4*, *SOX2*, *SV40T*, *KLF4* (OSTK, encoded by pEP4EO2SET2K). However, we failed to obtain stable iPS colonies from UCs or skin fibroblasts (Fig. 1F), suggesting that the OSTK four factor were insufficient for non-integrating iPS cell generation under serum-free conditions. We and several other groups had shown that miR-302-367 cluster could greatly enhance somatic reprogramming efficiency [24], [25], [26]. In addition, we found that mice chimeras with genome integration of miR-302-367 cluster and their offspring are tumors-free for over 2 years. Thus, miR-302-367 cluster might be less genomically toxic and even suppress tumorigenecity of human pluripotent stem cells [27] and be a better choice for iPS cells generation than *c-MYC*. We then constructed an episomal vector expressing human miR-302-367 cluster (named pCEP4-miR302-367 cluster. Fig. S2A and S2B) and simultaneously transfected it into UCs with OSTK through electroporation. The transfected UCs were then cultured on matrigel-coated plate and in totally defined and serum free medium (mTesR1 or E8) for reprogramming. Around 20 days after electroporation, we could observe human ES like colonies. The cells exhibited traditional human ES/iPS morphology with a high nuclear to cytoplasmic ratio (Fig. 1G). However, the reprogramming efficiencies varied a lot for different donors. For 5×10^5^ cells we routinely used to start reprogramming, the number of human ES like colonies varied between 0 and 72 for different individuals. The representative reprogramming efficiencies of three different donors were shown in Figure 1H and efficiencies of other donors were listed in Table 1. Most of the colonies with human ES like morphology could be picked and expanded successfully. To the contrary, when we applied the same reprogramming factors combination on skin fibroblasts, we did observe morphology change, but failed to obtain alkaline phosphatase (AP) positive iPS cell colonies under same culture condition (Fig. 1F). These data indicated that UCs were much easier to be reprogrammed than skin fibroblasts. This is consistent to our previous finding that the fibroblasts not the UCs need to go through a mesenchymal-to-epithelial transition (MET) process, which might hamper their iPS cell generation [28]. We further confirmed this by showing that UCs expressed higher level of markers or accelerators of MET, such as *E-CADHERIN*, *CLAUDIN*, *OCCLUDIN* and miR-200c, miR-302b, but lower level of repressors for MET, like *TWIST* (Fig. S2C). Moreover, we failed to generate human iPS cells from UCs with the episomal miR-302-367 cluster vector alone, consistent with a previous report [26].

**Figure 2:**
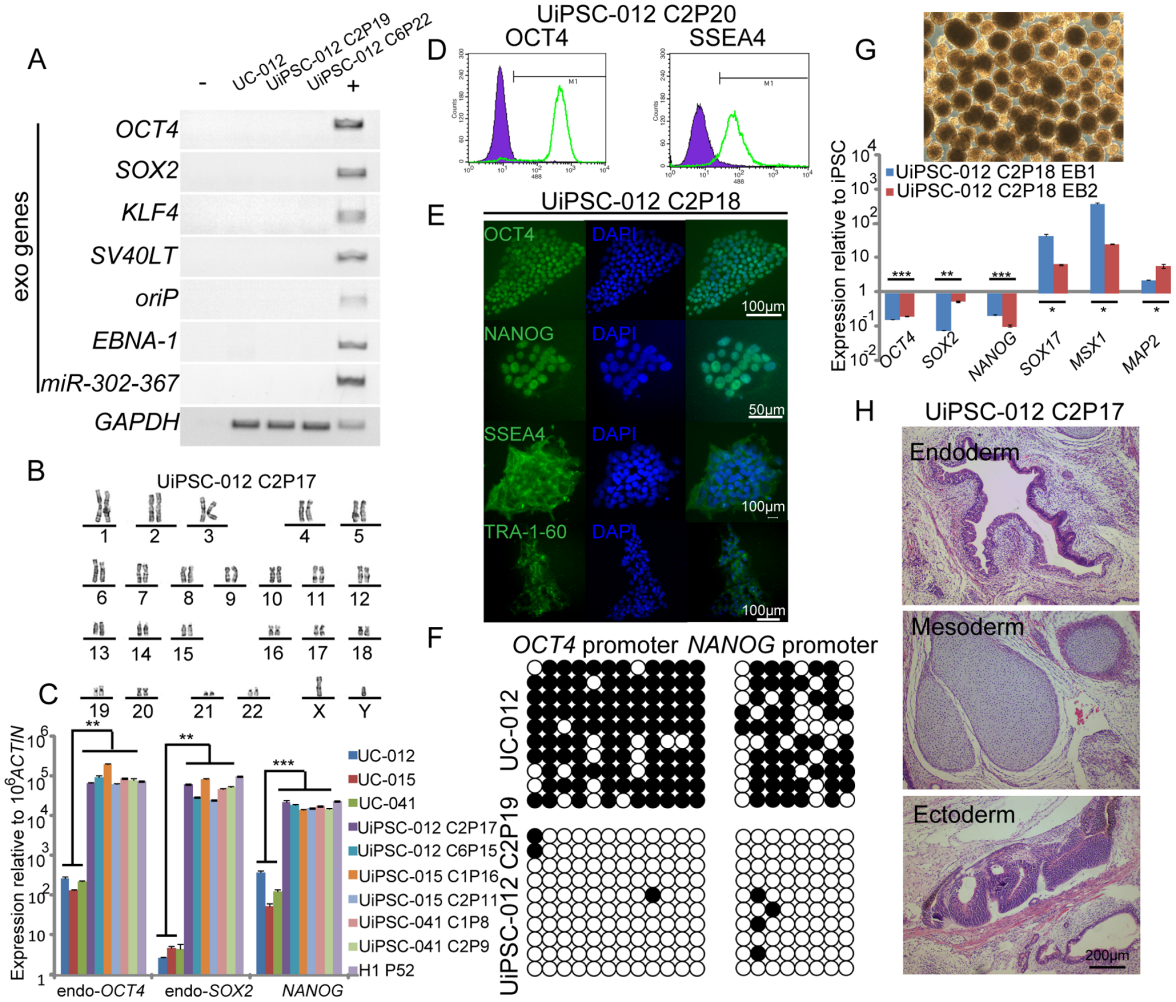
Characterization of a typical non-intergrated iPS cell line generated from UC-012. A. Non-integrating analysis of eipsomal DNA in the iPS cells. −: negative control. +: positive control, UCs transfected with indicated episomal vectors. UC-012: urine-derived cells 12. UiPSC-012 C2P19 and UiPSC-012 C6P22: iPS cell lines, C: colony number, P: passage. B. G-band analysis of the iPS cells derived from UC-012 shows normal karyotype. C. qPCR for endogenous human ES cell specific transcription factors of UC-012 and two derivative iPS cell lines. UC-015 and UC-041 and derivative iPS cell lines were also shown. Values were referred to 10^6^ copies of *ACTIN*. Human ES cell line H1 was used as control. n = 3. *P* value is referred to UCs. ** indicates *P*<0.01. D. Expression of human ES markers OCT4 and SSEA4 of UiPSC-012 C2P19 by flow cytometry. E. Immunofluorescence for human ES markers of UiPSC-012 C2P18. F. Methylation status of *OCT4* and *NANOG* promoters in UC-012 and UiPSC-012 C2P19. G. Top: EB formed from UiPSC-012 C2P18. Bottom: qPCR analysis of human ES cell markers and markers for the three germ layers relative to iPSCs. n = 3. * indicates *P*<0.05. H. HE-staining of the teratomas from UiPSC-012 C2P17.

To date, through the approaches described above, we have successfully generated UC derived iPS cells (UiPSCs) from 20 donors with different genetic and disease backgrounds (Table 1), demonstrating that it is a universal strategy, albeit with efficiencies varied for different donors. It is not surprise because the reprogramming efficiency variations had been well documented in mice [29], [30]. As for the donors, we haven’t found that the individuals with certain disease exhibited particularly different reprogramming efficiencies (listed in Table 1). The generation of iPS cells from UCs listed in Table 2 is underway. For each individual UC line, we usually picked and expanded at least 2 colonies for further characterization. Our standard iPS cell characterization was illustrated in Figure 2. The expanded colonies that passed the characterization including karyotyping, non-integrating and pluripotency will be deposited in the bank. Taking iPSCs generated from UC-012 for example, firstly, by using genomic PCR that could specifically amplify transgenes used for reprogramming, we confirmed that the stably expanded iPS colonies no longer harbored the exogenous reprogramming factors and episomal backbones (Fig. 2A), and kept the normal karyotype determined by G-band analysis (Fig. 2B). We further demonstrated that endogenous pluripotent genes such as *OCT4*, *SOX2* and *NANOG* were fully activated and were comparable to human embryonic stem cells (Fig. 2C–2E). Further by analyzing DNA methylation, we showed that the proximal promoters of *OCT4* and *NANOG* were de-methylated (Fig. 2F). We performed embryoid body (EB) formation assay to demonstrate that the iPS cells could form typical EBs that expressed genes of three germ layer lineages (Fig. 2G). We also tested the pluripotency *in vivo* through injection of iPS cells into immune-deficient mice (NOD-SCID) and demonstrated that they could generate teratomas containing three germ layer tissues (Fig. 2H). The information of characterization on other iPS cell lines was listed in Table 1 and Figure S3.

In all, we describe here a feasible and highly practical process to generate iPS cells from UCs in a totally defined condition that is feeder-free, virus-free and without using *c-MYC*. We have now generated 93 iPS cell lines from 20 individuals with various backgrounds (Table 1). Generation of non-viral and feeder-free iPS cell bank is an important step towards the further applications. Employing UCs for non-viral iPS cell generation offers several advantages over other cell types such as skin fibroblasts and blood cells. Firstly, it is a totally non-invasive and easy process that is applicable for virtually any candidate to donate tissue, particularly for children and those patients with certain diseases such as hemophilia. Secondly, UCs exhibit the epithelial phenotype and are much easier to be reprogrammed to iPS cells than mesenchymal skin fibroblasts, by circumventing the MET process. This is particularly important for using non-viral approaches to generate iPS cells, as the efficiencies of most reported non-viral approaches are very low. Indeed, we failed to obtain stably expanded iPS cells from skin fibroblasts using our approaches described above. Lastly, the whole process is serum-free and feeder-free which makes it feasible to generate clinical non-viral human iPS cells under current good manufacture practice (cGMP) conditions in future.

One important safety concern for iPS cells is the genomic stability [31], [32], [33], [34], [35], which has been reported in extended passaging of human ESCs [36]. However, Geti *et al*. reported recently that iPSCs process appeared to generate little or no CNVs, even using vial approaches [35]. Further studies will be needed to analyze the genomic and epigenomic stabilities in detail on the iPS cell lines generated from different genetic backgrounds. The non-integrating iPS cell bank described here contains dozens of iPS cell lines from different individuals but generated in the same laboratory and the same condition, thus provides a valuable source for comprehensive comparative analysis. The bank is expanding rapidly and also a good resource for disease modeling and future applications in regenerative medicine.

We have previously reported that UCs can be reprogrammed into neural progenitor cells (NPCs) through the same combination of vectors described in this report plus a cocktail of small molecules to inhibit signaling pathways [37]. It had been shown that the external signaling pathways such as SMAD and GSK3β signaling are important in shaping the neural stem cell fate [38], [39]. As for the urine cell reprogramming, we reasoned that the signaling inhibitors present in the medium played critical roles in determining whether the cells become NPCs or iPSCs. Our future work is to dissect the molecular mechanisms of the reprogramming process in the presence of those signaling inhibitors.

## Materials and Methods

### Ethical Statement

The individuals in this manuscript have signed written informed consent for donating human UCs for stem cell generation. The experiments involving human subject and animal research had been reviewed and approved by IRB at GIBH (NO. 2010012).

### Collection and Expansion of UCs

UCs were collected and cultured as described previously [13]. The cells passaged less than 4 times were used for iPS cell generation.

All patients with genetic diseases were diagnosed by clinical standards in our collaborated hospital. Sanger sequencing was used to confirm the potential mutations.

### EdU (5-ethynyl-2′-deoxyuridine) Imaging and Cell Proliferation Analysis

EdU labeling and cell proliferation analysis was performed using Click-iT® EdU Imaging Kit with Alexa Fluor® (Life technologies), following the instructions of the manufacturer. 1.5×10^4^ human UCs at passage 4 were seeded per well in 24-well plates. EdU was added to cells 12 h after seeding and pulsed for 2.5 h. The EdU-treated cells were fixed and followed by the highly-specific click reaction. A Leica DMI6000B microscope (Leica Microsystems GmbH, Germany) was used for observation and photographing. Samples were analyzed in triplicate.

### iPS Cell Generation

Briefly, 5×10^5^–1×10^6 ^UCs at early passages were individualized by trypsin treatment (0.25%Trypsin/0.5 mM EDTA, Gibco) and electroporated with indicated episomal plasmids using Amaxa™ Basic Nucleofector™ Kit for primary mammalian Epithelial cells, program T-020 (LONZA), or cells could be resuspended in PBS solution and electroporated using Gene Pulser MXcell Electroporation System (BIO-RAD). The electroporated UCs were seeded onto matrigel (BD) pre-coated P6 wells. In each electroporation, 3.5 µg pEP4EO2SET2K (contains *OCT4*, *SOX2*, *SV40LT* and *KLF4*) and 2.5 µg pCEP4-M2L (contains *c-MYC* and *LIN28*) or pCEP4-miR-302-367 cluster (contains miR-302b, c, a, d and miR-367) were used. Defined medium mTeSR1 (STEMCELL) or E8 medium (DMEM/F12 (Hyclone) +2% ITS (Gibco)+Vc (70 µg/ml, Sigma)+bFGF (100 ng/ml, Invitrogen)+Tgfβ1 (2 ng/ml, PeproTech) [22]) was changed daily during generation. The iPS cell colonies were picked at around Day 20-Day 30 and cultured in mTeSR1 or E8 on matrigel. The culture medium was changed daily. The iPS cells were passaged with dispase (Gibco, 1 mg/ml in DMEM/F12) for the initial 2–5 passages depending on the cells and then passaged with 0.5 mM EDTA (Sigma, dissolved in PBS (Gibco)).

### iPS Cells Characterization

PCR analysis of exogenous reprogramming factors and episomal backbone integration, immunofluorescence microscopy (OCT-3/4 Antibody, Santa Cruz Biotechnology sc-5279; Human Nanog Affinity Purified Polyclonal Ab, R&D AF1997; Anti-SSEA4 antibody, Abcam AB16287; Anti-TRA-1-60, Millipore MAB 4360), AP staining, karyotyping, bisulfate sequencing and teratoma formation were performed as we described previously [13], [37]. The primers used for cloning, PCR analysis, qPCR and bisulfate sequencing were listed in Table S3. Total RNA was extracted using Trizol (invitrogen), and reverse transcribed by oligo dT or specific primers. qPCR was performed in triplicate with CFX96 machine (BIO-RAD) and SYBR Green Premix EX Taq™ Kit (Takara) following the instructions by the manufacturer.

### Flow Cytometry Analysis

About 1×10^6^ iPS cells were used for each stanning. iPS cells were trypsinized and fixed with 1% paraformaldehyde dissolved in PBS for 10 min at 37°C. The cells were then washed with FACS buffer (PBS contains 2% fetal bovine serum, FBS) and resuspended in 90% ethanol for permeabilization by incubating 30 min on ice. After washing, cells were sequencially incubated with primary and secondary antibody for 30 min at 37°C. Control samples were stained with isotype-matched control antibodies. The iPS cells were washed and resuspended in FACS buffer and then processed for analysis on FACS Calibur (BD).

### Embryonic Body (EB) Formation and *in vitro* Differentiation

iPS colonies on matrigel coated plate were incubated with collagenase IV (Gibco) for about 30 min at 37°C till the colonies were completely dissociated. The colonies were washed with DMEM/F12 and resuspended in EB medium (DMEM/F12+20% Knockout serum replacement, KSR (Gibco) +1% L-GlutaMax (Gibco) +1% NEAA (Gibco) +1% β-mercaptoethanol (Gibco)). The colonies were cultured in suspension for 4 days then transferred onto matrigel coated plate and cultured for 4 days more.

### Statistical Analyses

Data were shown in mean. Error bars indicated standard deviation of the mean. *P* values were calculated by one-way ANOVA and Bonferroni post-hoc test, or student’s t test by Origin.

### Author Declaration

Cell lines and samples related in this report are available upon request for noncommercial use.

## Supporting Information

Figure S1 **A. Anonymous survey results of willingness for donating samples.** B. Mutation of *HBB* in UC-041 (β-thalassemia carrier) and UiPSC-041 C1P8 by sequencing. C. Mutations of *HBB* in UC-009 (β-thalassemia) and UiPSC-009 C3P15 by sequencing.
(TIF)

Figure S2 **A. Map of the plasmid pCEP4-hsa-miR-302-367 cluster used in the study.** B. qPCR for miR-302b expression of cells transfected with plasmid pCEP4-has-miR-302-367 cluster. Cells transfected with pCEP4-EGFP were used as control. Values are referred to 10^6^ copies of *RNU6*. C. qPCR for MET related genes expression of UCs relative to skin fibroblasts.
(TIF)

Figure S3 **Characterization of two typical non-intergrating iPS cell lines generated from UC-015 and UC-041 respectively.** A. Non-integrating analysis of eipsomal DNA in the iPS cell lines generated from UC-015. B. G-band analysis of UiPSC-015 C1P12. C. Immunofluorescence for human ES markers of UiPSC-015 C1P16. D. Methylation status of *OCT4* and *NANOG* promoters in UC-015 and UiPSC-015 C1P11. E. HE-staining of the teratomas from UiPSC-015 C1P16. F. Non-integrating analysis of eipsomal DNA in the iPS cell lines generated from UC-041. G. G-band analysis of UiPSC-041 C1P9. H. Immunofluorescence for human ES markers of UiPSC-041 C1P12. I. Methylation status of *OCT4* and *NANOG* promoters in UC-041 and UiPSC-041 C1P8. J. HE-staining of the teratomas from UiPSC-041 C1P10.
(TIF)

Table S1 **Mutation detection of UC-001(Alport’s syndrome).**
(DOCX)

Table S2 **Mutation detection of UC-044 (ALS).**
(DOCX)

Table S3 **Primer list.**
(DOCX)
