# Supplementary figures and images for: Generating a Non-Integrating Human Induced Pluripotent Stem Cell Bank from Urine-Derived Cells
Source: PLoS One. 2013 Aug 5;8(8):e70573. doi: 10.1371/journal.pone.0070573 (PMC3734275; doi:10.1371/journal.pone.0070573)

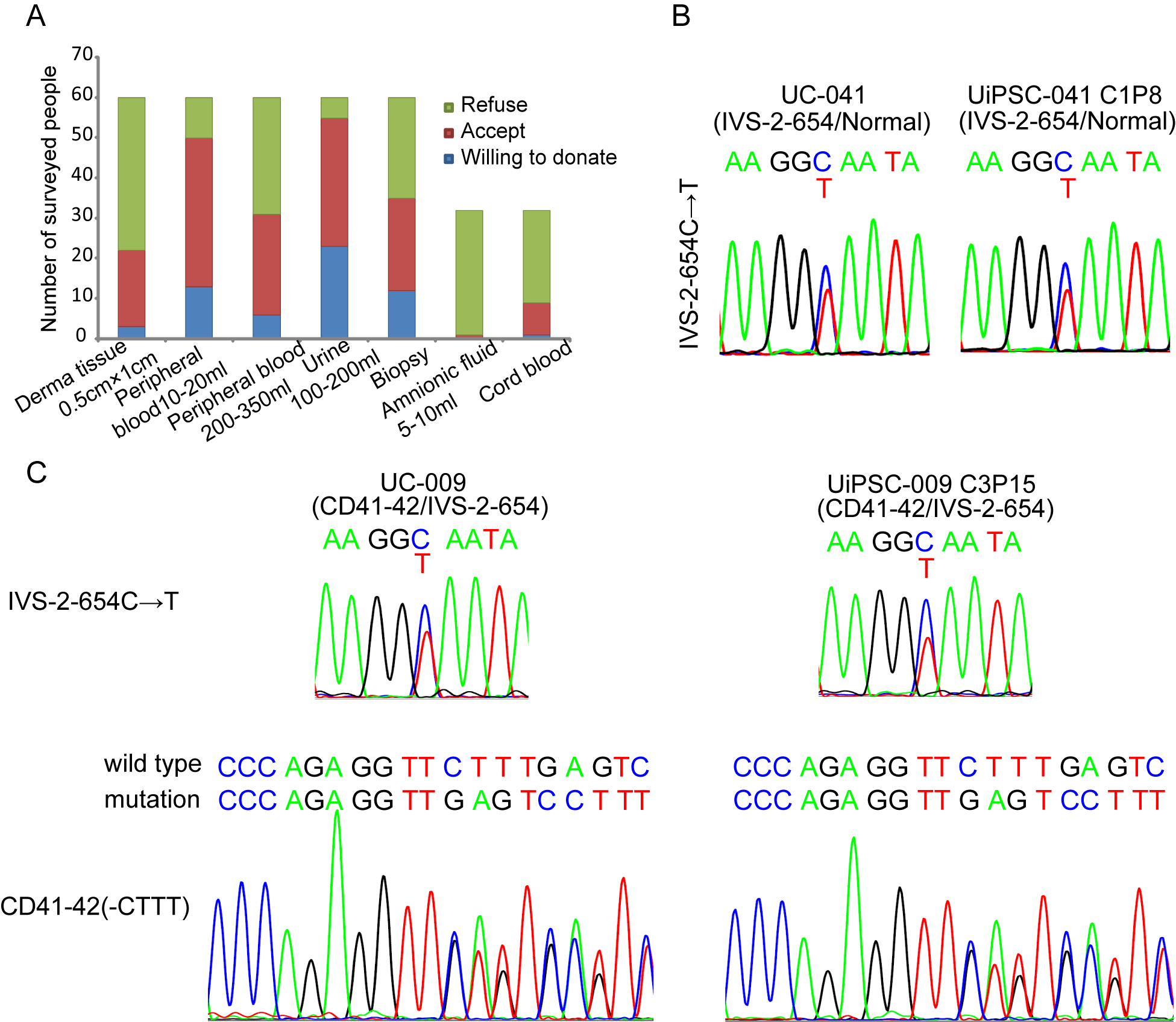

Supplement: Figure S1 — A. Anonymous survey results of willingness for donating samples. B. Mutation of HBB in UC-041 (β-thalassemia carrier) and UiPSC-041 C1P8 by sequencing. C. Mutations of HBB in UC-009 (β-thalassemia) and UiPSC-009 C3P15 by sequencing. (TIF) [file pone.0070573.s001.tif]

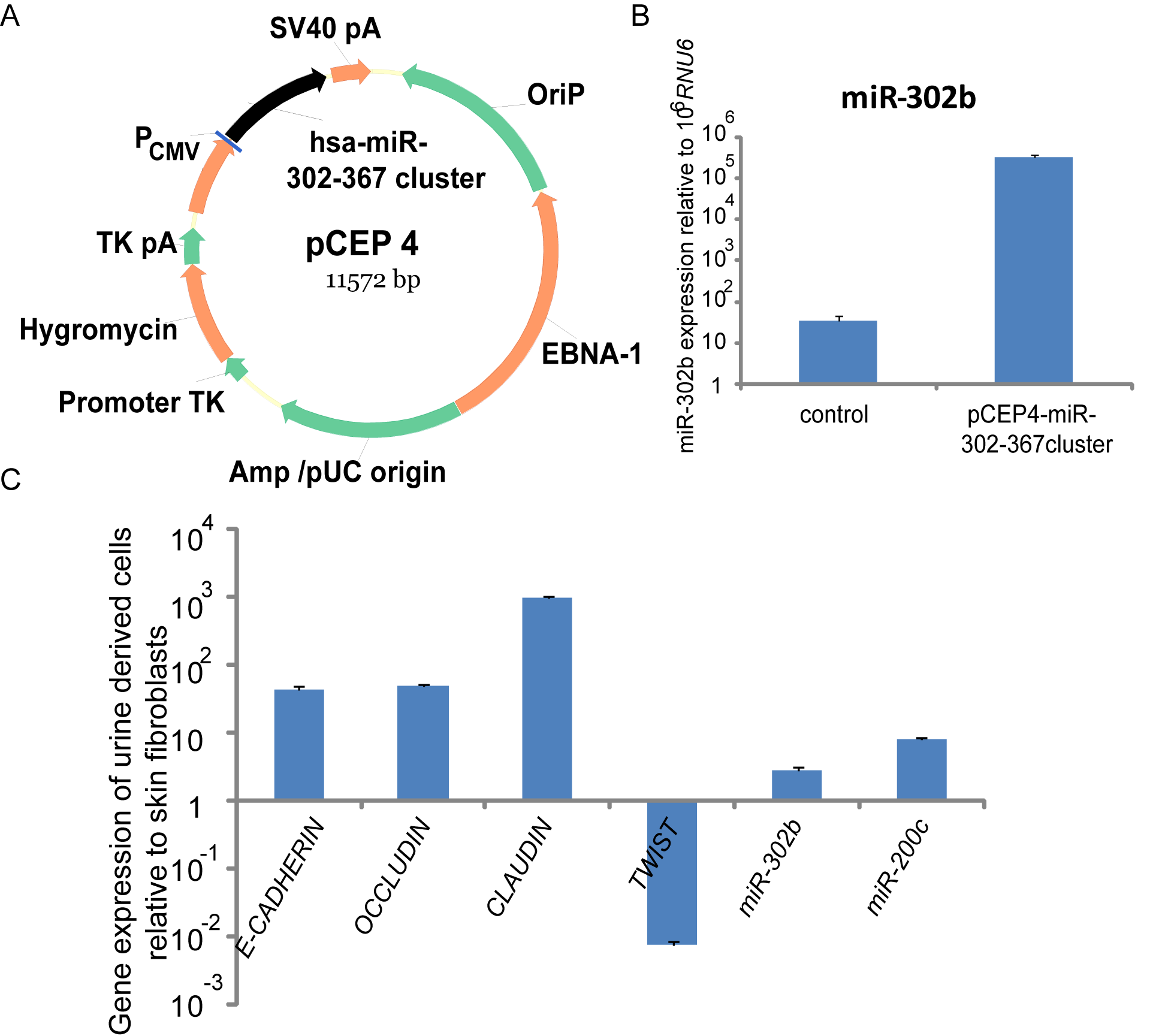

Supplement: Figure S2 — A. Map of the plasmid pCEP4-hsa-miR-302-367 cluster used in the study. B. qPCR for miR-302b expression of cells transfected with plasmid pCEP4-has-miR-302-367 cluster. Cells transfected with pCEP4-EGFP were used as control. Values are referred to 106 copies of RNU6. C. qPCR for MET related genes expression of UCs relative to skin fibroblasts. (TIF) [file pone.0070573.s002.tif]

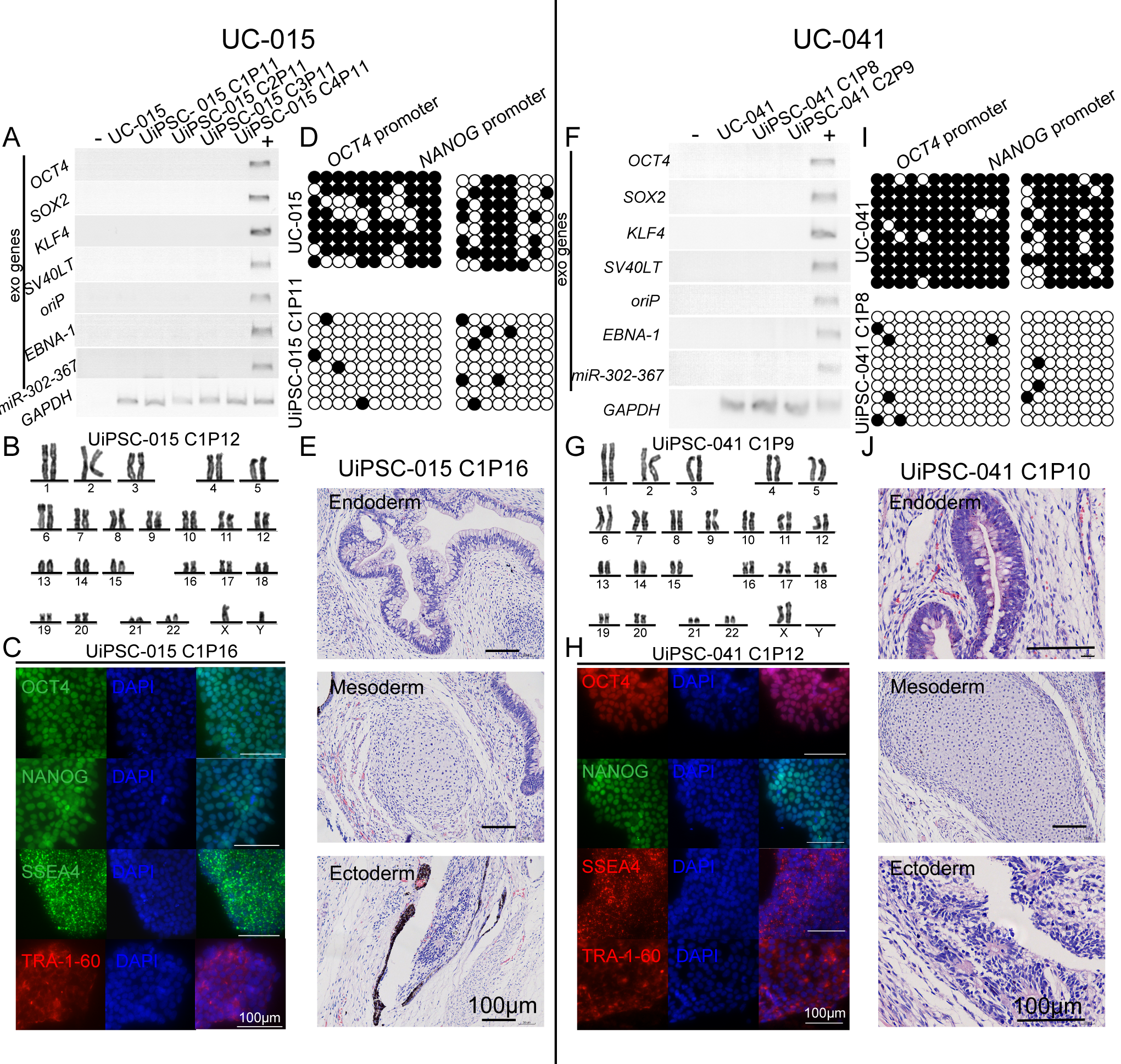

Supplement: Figure S3 — Characterization of two typical non-intergrating iPS cell lines generated from UC-015 and UC-041 respectively. A. Non-integrating analysis of eipsomal DNA in the iPS cell lines generated from UC-015. B. G-band analysis of UiPSC-015 C1P12. C. Immunofluorescence for human ES markers of UiPSC-015 C1P16. D. Methylation status of OCT4 and NANOG promoters in UC-015 and UiPSC-015 C1P11. E. HE-staining of the teratomas from UiPSC-015 C1P16. F. Non-integrating analysis of eipsomal DNA in the iPS cell lines generated from UC-041. G. G-band analysis of UiPSC-041 C1P9. H. Immunofluorescence for human ES markers of UiPSC-041 C1P12. I. Methylation status of OCT4 and NANOG promoters in UC-041 and UiPSC-041 C1P8. J. HE-staining of the teratomas from UiPSC-041 C1P10. (TIF) [file pone.0070573.s003.tif]
