# Supplementary material for: Generating a Non-Integrating Human Induced Pluripotent Stem Cell Bank from Urine-Derived Cells
Source: PLoS One. 2013 Aug 5;8(8):e70573. doi: 10.1371/journal.pone.0070573 (PMC3734275; doi:10.1371/journal.pone.0070573)
Supplement: Table S1 — Mutation detection of UC-001(Alport’s syndrome). (DOCX) [file pone.0070573.s004.docx]

| **Gene symbol** | **Mutation** | **Result** |
| --- | --- | --- |
| COL4A5 | genome 237657-247851 deletion | No mutation |
| COL4A5 | GLY1143ASP | No mutation |
| COL4A5 | GLY325ARG | No mutation |
| COL4A5 | GLY521CYS | No mutation |
| COL4A5 | GLY325GLU | No mutation |
| COL4A5 | GLY54ASP | No mutation |
| COL4A5 | ARG1677GLN | No mutation |

Table S1: Mutation detection of UC-001(Alport’s syndrome).
