# Supplementary material for: Generating a Non-Integrating Human Induced Pluripotent Stem Cell Bank from Urine-Derived Cells
Source: PLoS One. 2013 Aug 5;8(8):e70573. doi: 10.1371/journal.pone.0070573 (PMC3734275; doi:10.1371/journal.pone.0070573)
Supplement: Table S2 — Mutation detection of UC-044 (ALS). (DOCX) [file pone.0070573.s005.docx]

| **Gene symbol** | **Mutation** | **Result** | **Gene symbol** | **Mutation** | **Result** |
| --- | --- | --- | --- | --- | --- |
| SOD1 | A4V | No mutation | FUS | R216C | No mutation |
| SOD1 | H46R | No mutation | FUS | G225V | No mutation |
| SOD1 | G93A | No mutation | FUS | G230C | No mutation |
| SOD1 | L144F | No mutation | FUS | R234C/L | No mutation |
|  | | | FUS | R244C | No mutation |
| TDP43 | G287S | No mutation | FUS | S402_P411delinsGGGG | No mutation |
| TDP43 | G290S | No mutation |  |  |  |
| TDP43 | S292N | No mutation |  |  |  |
| TDP43 | G294A/V | No mutation | FUS | S462F | No mutation |
| TDP43 | G295R | No mutation | FUS | G466VfsX14 | No mutation |
| TDP43 | D169G | No mutation | FUS | R495X | No mutation |
| TDP43 | G298S | No mutation | FUS | G509D | No mutation |
| TDP43 | M311V | No mutation | FUS | K510R/E | No mutation |
| TDP43 | A315T | No mutation | FUS | S513P | No mutation |
| TDP43 | A321V/G | No mutation | FUS | R514G/S | No mutation |
| TDP43 | Q331K | No mutation | FUS | R514S;G515C | No mutation |
| TDP43 | S332N | No mutation | FUS | H517Q/P | No mutation |
| TDP43 | G335D | No mutation | FUS | R518G/K | No mutation |
| TDP43 | M337V | No mutation | FUS | R521G/H | No mutation |
| TDP43 | Q343R | No mutation | FUS | R522G | No mutation |
| TDP43 | N345K | No mutation | FUS | R524W/T/S | No mutation |
| TDP43 | G348C/V | No mutation | FUS | P525L | No mutation |
| TDP43 | N352T/S | No mutation |  |  |  |
| TDP43 | R361S | No mutation | ANG | A107T | No mutation |
| TDP43 | P363A | No mutation | ANG | A122T | No mutation |
| TDP43 | Y374X | No mutation | ANG | A121G | No mutation |
| TDP43 | N378D | No mutation | ANG | G164A | No mutation |
| TDP43 | S379P/C | No mutation | ANG | C189G | No mutation |
| TDP43 | A382P/T | No mutation | ANG | A191T | No mutation |
| TDP43 | I383V | No mutation | ANG | A208G | No mutation |
| TDP43 | G384R | No mutation |  |  |  |
| TDP43 | N390D/S | No mutation | OPTN | exon5 AGACCAGCCTGG deletion | No mutation |
| TDP43 | S393L | No mutation |  |  |  |
|  | | |  |  |  |
| FUS | S57del | No mutation |  |  |  |
| FUS | G156E | No mutation | OPTN | Q398X | No mutation |
| FUS | G187S | No mutation | OPTN | E478G | No mutation |
| FUS | G191S | No mutation |  |  |  |
|  |  |  |  |  |  |

Table S2: Mutation detection of UC-044 (ALS).
