# Supplementary material for: Generating a Non-Integrating Human Induced Pluripotent Stem Cell Bank from Urine-Derived Cells
Source: PLoS One. 2013 Aug 5;8(8):e70573. doi: 10.1371/journal.pone.0070573 (PMC3734275; doi:10.1371/journal.pone.0070573)
Supplement: Table S3 — Primer list. (DOCX) [file pone.0070573.s006.docx]

Table S3: Primer list

| **Genes** | **Size(bp)** | **Symbol** | **Sequence(5'to3')** |
| --- | --- | --- | --- |
| ***For gene cloning*** | | | |
| *miR-302/367* | 926 | PFhV4-302 | CTAGCTAGCGAACACGAATCTTTGGGAAC |
|  |  | PRhV4-302 | CCGTCTCGAGCATAAAGAGAGACATAAAATGGG |
| ***For qPCR*** | | | |
| *ACTIN** | 365 | Q-ACTIN-F | CCCAGAGCAAGAGAGG |
|  |  | Q-ACTIN-R | GTCCAGACGCAGGATG |
| *endoOCT4** | 164 | endoOCT4-F | CCTCACTTCACTGCACTGTA |
|  |  | endoOCT4-R | CAGGTTTTCTTTCCCTAGCT |
| *endoSOX2** | 151 | endoSOX2-F | CCCAGCAGACTTCACATGT |
|  |  | endoSOX2-R | CCTCCCATTTCCCTCGTTTT |
| *NANOG* | 237 | hNanog-q2-F | AAGGTCCCGGTCAAGAAACAG |
|  |  | hNanog-q2-R | CTTCTGCGTCACACCATTGC |
| *SOX17* | 152 | Q-SOX17F | ACCGCACGGAATTTGAAC |
|  |  | Q-SOX17R | GCAGTAATATACCGCGGAGC |
| *MSX1* | 90 | Q-MSX1F | TCCGCAAACACAAGACGA |
|  |  | Q-MSX1R | ACTGCTTCTGGCGGAACTT |
| *MAP2* | 129 | Q-MAP2F | TGAAGCAAAGGCACCTCAC |
|  |  | Q-MAP2R | TATGGGAATCCATTGGCG |
| ***For PCR*** | | | |
| *OCT4*** | 657 | Oct4-SF1 | AGTGAGAGGCAACCTGGAGA |
|  |  | IRES2-SR | AGGAACTGCTTCCTTCACGA |
| *SOX2*** | 534 | Sox2-SF1 | ACCAGCTCGCAGACCTACAT |
|  |  | SV40pA-R | CCCCCTGAACCTGAAACATA |
| *KLF4*** | 401 | Klf4-SF1 | CCCACACAGGTGAGAAACCT |
|  |  | SV40pA-R | CCCCCTGAACCTGAAACATA |
| *SV40LT*** | 491 | SV40T-SF1 | TGGGGAGAAGAACATGGAAG |
|  |  | IRES2-SR | AGGAACTGCTTCCTTCACGA |
| *oriP*** | 544 | pEP4-SF1 | TTCCACGAGGGTAGTGAACC |
|  |  | pEP4-SR1 | TCGGGGGTGTTAGAGACAAC |
| *EBNA-1*** | 666 | pEP4-SF2 | ATCGTCAAAGCTGCACACAG |
|  |  | pEP4-SR2 | CCCAGGAGTCCCAGTAGTCA |
| *miR-302/367* | 322 | pCEP4-1627F2 | TTTCCAAAATGTCGTAATAACCCCG |
|  |  | pCEP4-C1306R | CTCCCAAAGAGTCCTGTTCTGTCCT |
| *Gapdh*** | 152 | GAPDH-F | GTGGACCTGACCTGCCGTCT |
|  |  | GAPDH-R | GGAGGAGTGGGTGTCGCTGT |
| ***For bisulfite-sequencing PCR*** | | | |
| *OCT4* | 767 | Hs Oct4 pro 1410F | AGGTGTGGGAGTGATTTTAGATAGT |
|  |  | Hs Oct4 pro R4 | AAACCTTAAAAACTTAACCAAATC |
|  | 467 | mehOCT4 F2-S | GAGGTTGGAGTAGAAGGATTGTTTTGG |
|  |  | mehOCT4 F2-AS | CCCCCCTAACCCATCACCTCCACCACC |
| *NANOG* | 528 | Hs Nanogpro1444F | TTGTTGTTTAGGTTGGAGTATAGTGG |
|  |  | Hs Nanogpro1971R | CCTAACGAACACACCCCCTACT |
|  | 336 | mehNANOG-F1-S | TGGTTAGGTTGGTTTTAAATTTTTG |
|  |  | mehNANOG-F1-AS | AACCCACCCTTATAAATTCTCAATTA |

*The sequences for the primers used to amplify these products are taken from ref. 14.

**The sequences for the primers used to amplify these products are taken from ref. 15.
